# ﻿Five new synonyms for *Impatiensprocumbens* (Balsaminaceae) in China

**DOI:** 10.3897/phytokeys.222.97087

**Published:** 2023-03-29

**Authors:** Rong-Xin Huang, Tao-Hua Yuan, Yi Chen, Mei-Jun Li, Xin-Xiang Bai

**Affiliations:** 1 College of Forestry, Guizhou University CN-550025 Guiyang, Guizhou, China Guizhou University Guiyang China; 2 Wuhan, China unaffiliated Wuhan China; 3 Qianxinan Academy of Agricultural and Forestry Sciences, CN-562400 Xingyi, Guizhou, China Qianxinan Academy of Agricultural and Forestry Sciences Xingyi China

**Keywords:** Lectotypification, morphology, taxonomy

## Abstract

In the revision on the genus *Impatiens* L. in China, we found that there were synonyms amongst some species. *Impatiensprocumbens* Franch. morphologically resembled *I.reptans* Hook.f., *I.crassiloba* Hook.f., *I.ganpiuana* Hook.f., *I.atherosepala* Hook.f. and *I.rhombifolia* Y.Q.Lu & Y.L.Chen. After a thorough morphological study, based on original literature, type specimens and field surveys, it was found that the above six species of *Impatiens* had no substantial differences in morphological characters and there was continuity in geographical distribution. Therefore, we determined that *I.reptans*, *I.crassiloba*, *I.ganpiuana*, *I.atherosepala* and *I.rhombifolia* are the synonyms of *I.procumbens*. At the same time, we present the color photographs, supplementary descriptions of morphology, and geographical distribution. The lectotype of *I.procumbens* and *I.reptans* are also designated here.

## ﻿Introduction

In China, 352 species of *Impatiens* have been recorded, including 273 species endemic to China, which are concentrated throughout the Qinling Mountains, southern Tibet, the Hengduan Mountains, Yunnan–Guizhou–Guangxi karst region, the middle and lower reaches of the Yangtze River and other regions (Yuan et al. 2022). It is well known that *Impatiens* species are notoriously difficult to identify, because of their abundant character variations and morphological similarities, which makes the boundary between species very blurred (Grey-Wilson 1980; Cong 2007; Tian et al. 2007). From the perspective of research history, there were many factors in the early published species, such as the distance between collectors and researchers, the collection of specimens with the same number in different herbaria, the change of place names etc., all of which have led to taxonomic problems, for example, the same species with different names, the absence of characters, and incorrect records. Additionally, these classification problems have not been resolved so far, which is not conducive to the research of *Impatiens*, but also causes some obstacles in the research for identification of the genus *Impatiens* in China. *I.procumbens* was published by Franchet (1886) on the basis of specimens collected from Dali, Yunnan Province. *I.reptans*, *I.crassiloba*, *I.ganpiuana*, and *I.atherosepala* were described by J. D. Hooker, based on specimens collected from Guizhou Province by E. M. Bodinier and J. P. Cavalerie (Hooker 1908a, b). *I.rhombifolia* was published by Chen and Lu (1990), based on specimens collected from Mount Emei, Sichuan Province. Yu (2008) mentioned that *I.reptans*, *I.ganpiuana*, *I.procumbens* and *I.rhombifolia* were very similar in morphology and may be described repeatedly. Through extensive field investigations and textual research of specimens, it was found that there were no essential differences amongst the above six species. In addition, the guidelines and recommendations of Article 9 of the ICN (Turland et al. 2018) have been followed while designating the lectotype.

## ﻿Materials and methods

For morphological comparisons, we reviewed the original literature and related records, including the original literature description of each species, information of type specimens, synonyms and geographical distribution. The main sources of original literature are from Tropicos (http://www.tropicos.org), IPNI (http://www.ipni.org) and other websites. Otherwise, we also critically checked type specimens or high-resolution images of specimens involved in this study in BM, E, K, P, NY, WU, PE, IBSC, IBK, KUN, HGAS, GZAC, GZTM, SWFC and conducted fieldwork of type localities in Sichuan, Guizhou and Yunnan Provinces. We have obtained more than 10 field collections. Herbarium specimens were chosen carefully. We dissected the flowers of the plant in the field or in the habitat itself after collection of the plants. And various morphological characters, such as leaf size and shape, inflorescence type, flower color, etc., were carefully observed, measured and quality photographs were taken.

## ﻿Results and discussion

**Figure 1.**
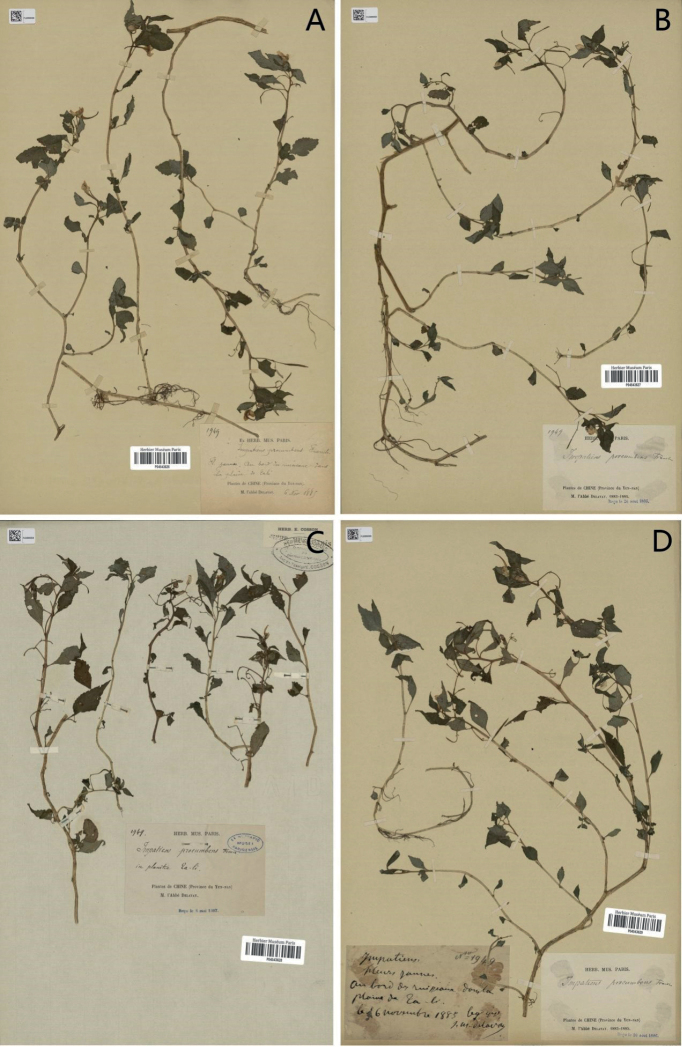
*Impatiensprocumbens***A** lectotype (P04543626) **B–D** isolectotype (P04543629, P04543628, P04543627). Source from http://coldb.mnhn.fr/catalognumber/mnhn/p/p04543626 (accessed on 1 September 2022). Source from http://coldb.mnhn.fr/catalognumber/mnhn/p/p04543629 (accessed on 1 September 2022). Source from http://coldb.mnhn.fr/catalognumber/mnhn/p/p04543628 (accessed on 1 September 2022). Source from http://coldb.mnhn.fr/catalognumber/mnhn/p/p04543627 (accessed on 1 September 2022).

**Figure 2.**
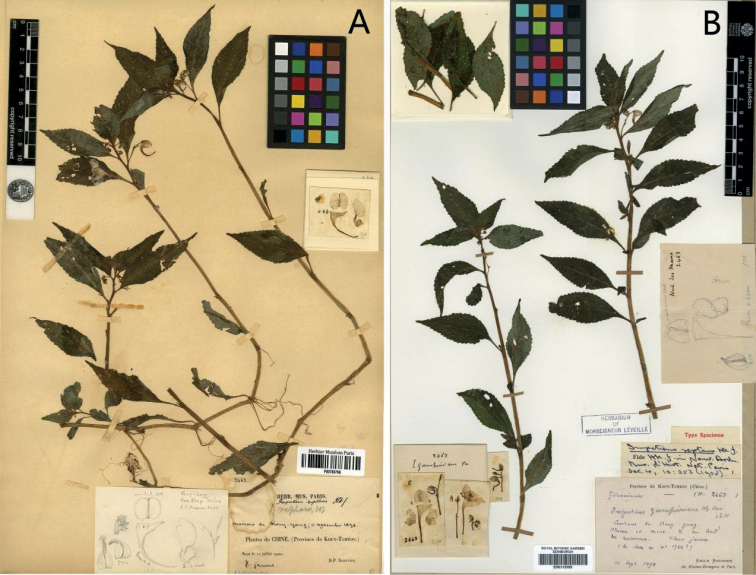
*Impatiensreptans***A** lectotype (P00780766) **B** isolectotype (E00313595). Source from http://coldb.mnhn.fr/catalognumber/mnhn/p/p00780766 (accessed on 8 April 2021). Source from https://data.rbge.org.uk/herb/E00313595 (accessed on 8 April 2021).

After consulting the type specimens (Fig. 1) of *Impatiensprocumbens* from Dali, Yunnan, *I.reptans* from Guiyang, Guizhou (Fig. 2) and *I.rhombifolia* from Emei, Sichuan (Fig. 4B), as well as the specimens in domestic and foreign herbaria and relevant literature records, it was found that there were no obvious differences in morphology amongst them (Table 1).

**Table 1.** Comparison of morphological characters of *Impatiensprocumbens*, *I.reptans*, *I.crassiloba*, *I.ganpiuana*, *I.atherosepala* and *I.rhombifolia* (data from protologue and type specimen).

Characters	*I.procumbens*	*I.reptans*	*I.crassiloba*	*I.ganpiuana*	*I.atherosepala*	*I.rhombifolia*
Stem	prostrate or procumbent	prostrate	prostrate	prostrate	no record	prostrate
Leaf	ovate or ovate-lanceolate, margin serrulate, 2–3 cm long, 1–1.5 cm wide, petiole 5–10 mm long	ovate or ovate-elliptic, margin crenate-serrate or serrate, 5–7 cm long, 2.5–3.5 cm wide, petiole 1–2 cm long	ovate, narrowly ovate or ovate-lanceolate, margin coarsely serrate, 2–7 cm long, 1–3 cm wide, petiole 5–20 mm long	elliptic, narrowly elliptic or lanceolate, margin coarsely serrate, 3–6 cm long, 1.5–3.5 cm wide, petiole 1–2 cm long	lanceolate, margin spinescent-serrate, 4–6 cm long, 1.5–2 cm wide, petiole 1–2 cm long	rhombic or subrhombic, margin serrate, 2–5 cm long, 1.2–1.6 cm wide, petiole 5–10 mm long
Lateral veins	5–6	6–7	6–8	4–6	6–8	5–6
Flower	1–2-flowered, 1.5–2 cm deep	2–3-flowered, ca. 2.5 cm deep	1–3-flowered, ca. 2.5 cm deep	1- or 2-flowered, ca. 2 cm deep	1-flowered, ca. 1.1 cm deep	2-flowered, ca. 1.5 cm wide
Lateral sepal	obliquely ovate, subfalcate, 3-veined, apex long cuspidate, 3–4 mm long	falcate-ovate, 3-veined, midvein apically stoutly mucronulate, 3–5 mm long	lanceolate, small, 3-5-veined, apex cuspidate, 3–5 mm long	ovate, abaxial midvein narrowly carinate, 3–4 mm long	ovate, aristate, arista ca. as long as sepals, 3-veined, ca. 5 mm long	yellow-green, ovate-orbicular, small, apex acute, ca. 5 mm long
Dorsal petal	orbicular, abaxial midvein thickened, narrowly carinate, 8–10 mm in diam.	orbicular, abaxial midvein fine, narrowly carinate, ca. 15 mm in diam.	orbicular, abaxial midvein carinate, ca. 10 mm in diam.	elliptic, abaxial midvein rostellate, carinate, ca. 10 mm long, ca. 8 mm wide	orbicular, abaxial midvein cristate, ca. 8 mm in diam.	orbicular, large, abaxial midvein thickened, ca. 15 mm long, ca. 13 mm wide
Lateral united petals	ca. 17 mm long, basal lobes subtetragonous, distal lobes obovate-oblong	ca. 22 mm long, basal lobes orbicular, small; distal lobes dolabriform	ca. 16 mm long, basal lobes orbicular, small; distal lobes large, apex 2-lobed	ca. 18 mm long, basal lobes broadly ovate; distal lobes dolabriform	ca. 12 mm long, basal lobes orbicular; distal lobes dolabriform, longer	ca. 18 mm long, basal lobes red spotted, orbicular, distal lobes dolabriform
Lower sepal	navicular, narrowed into an incurved spur, ca. 20 mm long	subnavicular, narrowed into an incurved spur, acute or divaricate, ca. 20 mm long	navicular, narrowed into a curved, long spur, ca. 18 mm long	funnelform, narrowed into an incurved spur, ca. 10 mm long	navicular, abruptly contracted into an incurved spur, ca. 15 mm long	navicular, narrowed into an erect spur; spur ca. 18 mm long
Anther	acute	acute	obtuse	obtuse	acute	acute

**Figure 3.**
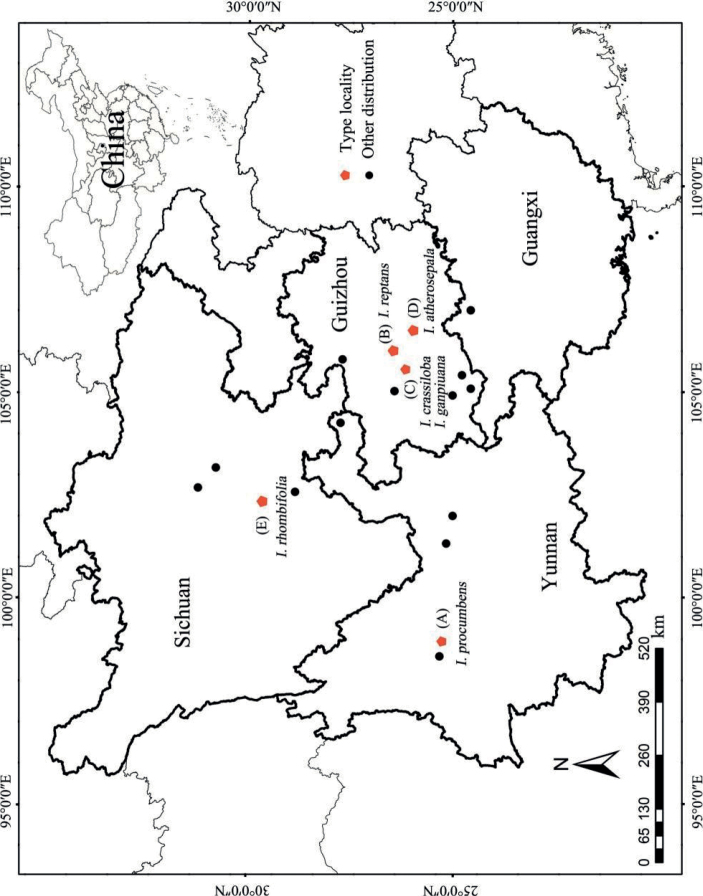
Geographical distributions **A** Dali City: the type locality of *Impatiensprocumbens***B** Guiyang City: the type locality of *I.reptans***C** ganpin: the type locality of *I.crassiloba* and *I.ganpiuana***D** pinfa: the type locality of *I.atherosepala***E** mount Emei: the type locality of *I.rhombifolia*.

**Figure 4.**
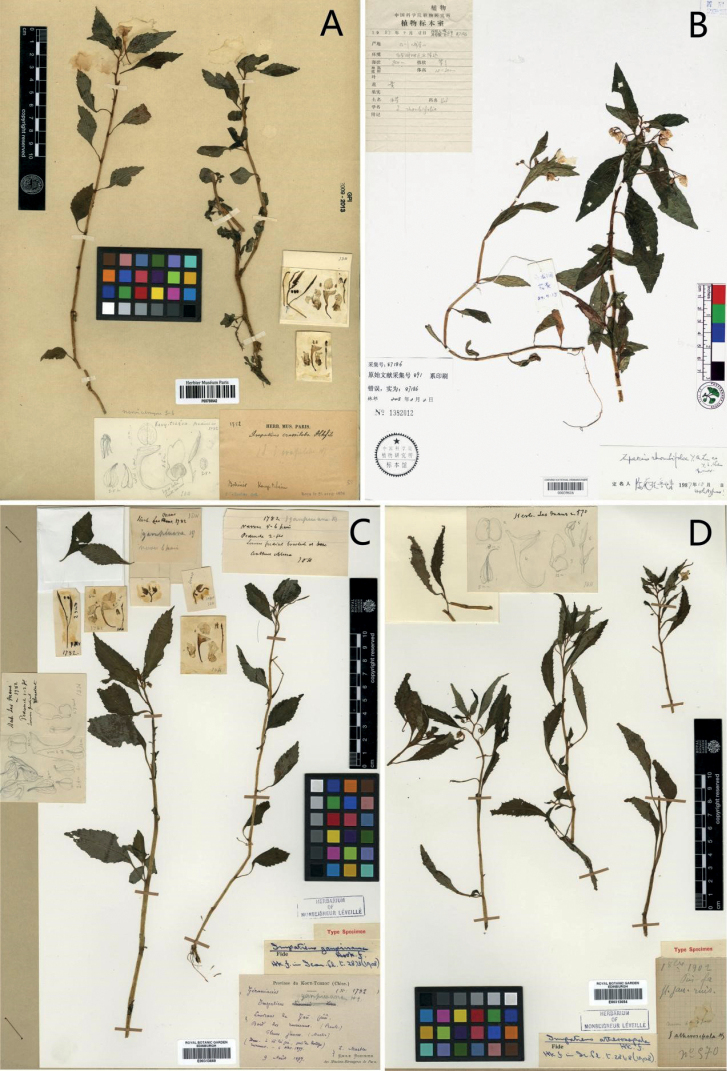
Type specimens **A***Impatienscrassiloba* (*E. M. Bodinier 1782*; P00780642) **B***I.rhombifolia* (*Lu YingQing 87186*; PE00039616) **C***I.ganpiuana* (*E. M. Bodinier 1782*; E00313669) **D***I.atherosepala* (*E. M. Bodinier 1782*; E00313654). Source from http://coldb.mnhn.fr/catalognumber/mnhn/p/p00780642 (accessed on 12 July 2021). Source from https://data.rbge.org.uk/herb/E00313669 (accessed on 20 March 2021). Source from https://data.rbge.org.uk/herb/E00313654 (accessed on 20 March 2021).

**Figure 5.**
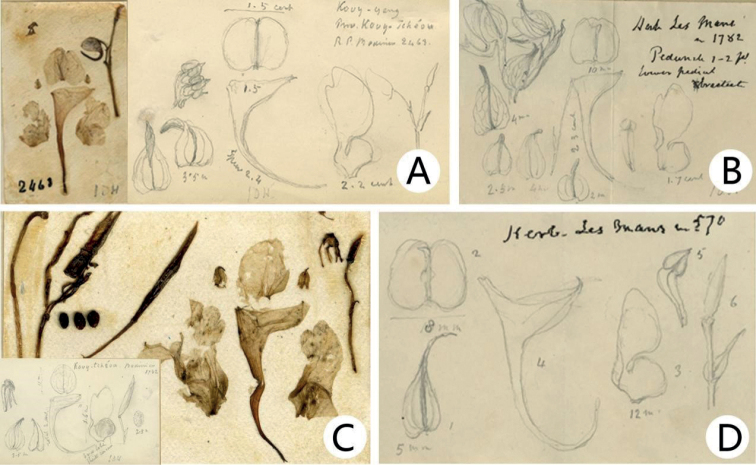
Flower dissected and drawing **A***Impatiensreptans* (on the part of E00313595 and P00780766) **B***I.ganpiuana* (on the part of E00313669) **C***I.crassiloba* (on the part of P00780642) **D***I.atherosepala* (on the part of E00313654). Source from http://coldb.mnhn.fr/catalognumber/mnhn/p/p00780766 (accessed on 8 April 2021). Source from https://data.rbge.org.uk/herb/E00313595 (accessed on 8 April 2021). Source from https://data.rbge.org.uk/herb/E00313669 (accessed on 20 March 2021). Source from http://coldb.mnhn.fr/catalognumber/mnhn/p/p00780642 (accessed on 12 July 2021). Source from https://data.rbge.org.uk/herb/E00313654 (accessed on 20 March 2021).

In the process of textual research on the type specimen of *Impatiensreptans* (Figs 2, 5A) , we found that J. D. Hooker did not designate a holotype in keeping with the practice of the time, the specimen P00780766 (Fig. 2A) agrees better with the information given in the literature and contains the pencil drawing of flower parts and the dissected flower parts pasted on the sheet. Therefore, it is designated here as the lectotype. We also found that the labelling information of the isolectotype (E00313595) was marked “An idem au no. 1782?”. At the same time, the vernacular name “*Impatiensganpiuana* Hf Vav” (Fig. 2B) was found on the label at the lower right corner. These two uncertainties point to the need for further textual research of *I.ganpiuana*. Meanwhile, the collection information marked on the type specimens of *I.crassiloba* (Fig. 4A) and *I.ganpiuana* (Fig. 4C) were the same, namely *E. M. Bodinier 1782*, the collection time of the latter being 9 August, 1897. Although there was no collection time on the specimen of the former, it could be inferred that the collection time may be from the same period according to the marked information that the specimens were received on 26 April 1898. Compared with the character description of the protologue, the differences mainly lay in the shape of the lower sepal and anther (Table 1), but by examining type specimens (Figs 4C, 5B), the shape of the lower sepal of *I.ganpiuana* should be cymbiform, so there was no obvious difference, except for the different anther.

**Figure 6.**
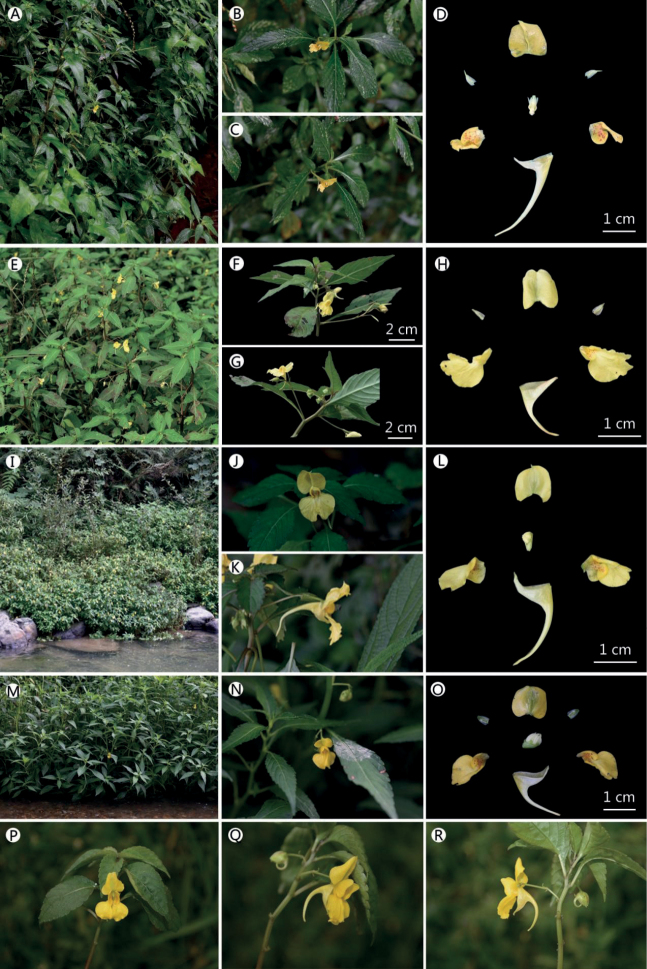
**A–D** population: Guiding County (type locality of *Impatiensatherosepala*: Pinfa) **A** habit **B** flower in face view **C** flower in lateral view **D** flower dissected **E–H** population: Mount Emei (type locality of *I.rhombifolia*) **E** habit **F** flower in face view **G** flower in lateral view **H** flower dissected **I–L** population: Kaiyang County (type locality of *I.reptans*: Guiyang) **I** habit **J** flower in face view **K** flower in lateral view **L** flower dissected **M–O** population: Guanshanhu District (near type locality of *I.crassiloba* and *I.ganpiuana*: ganpin) **M** habit **N** flower in face view **O** flower dissected **P–R** population: Dali City (type locality of *I.procumbens*) **P** flower in face view **Q, R** flower in lateral view. Photographs (**Q, R**) was taken in Dali City by QIN-WEN LIN, and other photographs by XIN-XIANG BAI.

**Figure 7.**
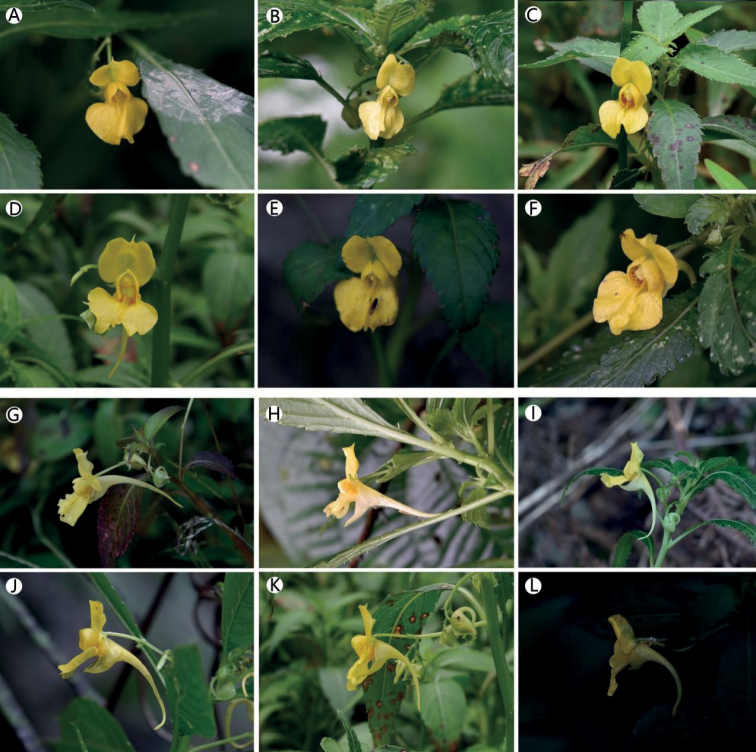
Floral variations (**A–F**) flower in face view **A** Guanshanhu District **B** Suiyang County **C** Huishui County **D** Zhenning County **E** Zhijin County **F** Guiding County **G–L** flower in lateral view **G** Danzhai County **H, I** Guiding County **J** Huishui County **K** Zhenning County **L** Zhijin County. Photographs by XIN-XIANG BAI.

At the same time, we found that *Impatiensatherosepala* is relatively close to the above plants of genus *Impatiens*, the type locality actually being Pingfa, Guiding County, which the “Flora of China” (Chen et al. 2007) misquoted as Pingba. From the type specimen (Figs 4D, 5D), it can be seen that the typical features are leaves lanceolate, margin with spinescent-serrate, 1-flowered inflorescence and lateral sepals ovate, with long aristate. The population we found in Pingfa, Guiding County (Fig. 6A–D) conformed to the description of these characters. Through extensive field investigations (Figs 6, 7), it was found that the leaf shape of genus *Impatiens* varied greatly and as a taxonomic character, it was not reliable. Meanwhile, the lateral sepals of *I.procumbens*, *I.reptans*, *I.crassiloba*, *I.ganpiuana*, *I.atherosepala* and *I.rhombifolia* had a tendency to extend and grow.

In addition, it can be seen from the type localities and other distribution points that the geographical distribution of these species is continuous and there is no obvious geographical isolation (Fig. 3).

It can be seen that there is no obvious difference in the morphology of the above six species and the geographical distribution is widespread. Since *Impatiensprocumbens* is the earliest name published, we concluded that *I.reptans*, *I.crassiloba*, *I.ganpiuana*, *I.atherosepala* and *I.rhombifolia* are the synonyms of *I.procumbens*. Franchet (1886) did not specify the type specimen when publishing the *I.procumbens*. Four specimens (collection no. 1949, collected by M. I’Abb é Delavy) made of flowering plants collected on 16 November 1885 are now in P (P04543629, P04543628, P04543627, P04543626), of which specimen P04543626 (Fig. 1A) is more consistent with the information described in the protologue, as well as containing complete plant organs and collection information. Therefore, P04543626 is designated as the lectotype here and P04543627, P04543628 and P04543629 are the isolectotypes.

### ﻿Taxonomic treatment

#### ﻿ Impatiens procumbens

Taxon classificationPlantaeEricales

Franch. in Bull. Soc. Bot. France 33: 447. 1886.

1CDF7073-CFF1-51FF-A357-C66079F37A29

Fig. 8

=Impatiensrhombifolia Y. Q. Lu & Y. L. Chen syn. nov. Type:–CHINA. Sichuan: Mt. Omei, 18 September 1987, *Yingqing Lu 87186* (holotype: PE [PE00039616]).
=Impatiensreptans Hook. f., syn. nov. Type:–CHINA. Guizhou: Guiyang, 11 September 1898, *E. M. Bodinier 2463* (lectotype, designated here: P [P00780766]; isolectotype: E [E00313595]).
=Impatienscrassiloba Hook. f., syn. nov. Type:–CHINA. Guizhou: Ganpin, without date, *E. M. Bodinier 1782* (holotype: P [P00780642]).
=Impatiensganpiuana Hook. f., syn. nov. Type:–CHINA. Guizhou: Ganpin, 09 August 1897, *E. M. Bodinier & L. Martin 2463* (holotype: E [E00313669]).
=Impatiensatherosepala Hook. f., syn. nov. Type:–CHINA. Guizhou: Pinfa, 01 October 1902, *J. P. Cavalerie & J. Pierre 570* (holotype: E [E00313654]).

##### Type.

China. Yunnan: Dali City, 6 November 1885, *M. I’Abbé Delavy 1949* (lectotype, designated here: P [P04543626]; isolectotype: P [P04543629, P04543628, P04543627]).

**Figure 8.**
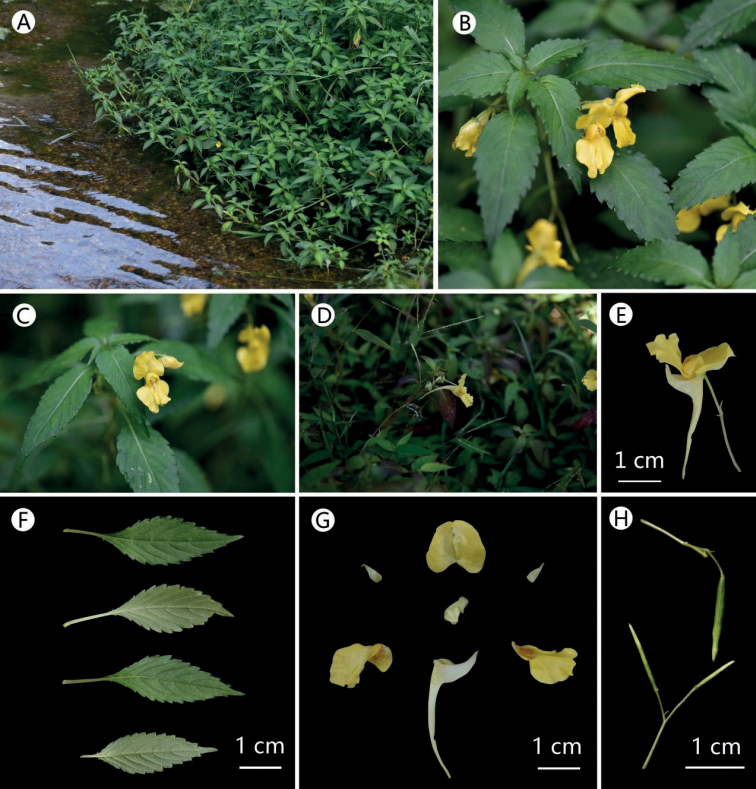
*Impatiensprocumbens***A** habit **B** plant **C** flower in face view **D, E** flower in lateral view **F** seed **G** leaf **H** flower dissected. Photographs by XIN-XIANG BAI.

##### Revised description.

Annual herbs, glabrous, 30–65 cm tall. Stem long prostrate basally, lower nodes with adventitious roots, green. Leaves alternate, lamina 4–7 cm long, 1–4 cm wide, lanceolate to ovate or ovate-elliptic, apex acuminate, margin crenate-serrate or spinescent-serrate, apex acuminate, base cuneate, blade dark green adaxially, pale green abaxially, herbaceous, lateral veins 6–9 pairs. Inflorescences axillary, racemose, 18–3 mm long, 1–3-flowered, bracteate base or above middle; bract 1, small, persistent, ovate-lanceolate, 3–4 mm long, 1–1.5 mm wide, herbaceous, green. Flower yellow, 2–3 cm deep, 1.5–2.5 cm wide. Lateral sepals 2, obliquely ovate, 4–6 mm long, 2–3 mm wide, abaxial mid-vein slightly thickened, green, herbaceous, 3-veined. Lower sepal cymbiform, 2–3.5 cm deep (including spur), mouth vertical, apex slightly acute, narrowed into a long spur, 1.5–2 cm long. Dorsal sepal orbicular, ca. 1 cm in diam., base cordate, apex retuse, abaxial midvein fine, narrowly carinate. Lateral united petals 2-lobed, not clawed, basal lobes small, orbicular, ca. 3 mm long, ca. 2 mm wide, with red spots; distal lobes dolabriform, ca. 12 mm long, ca. 6 mm wide, with dorsal inflexed auricle and notch in subapical portion on dorsal side. Filaments short, anther acute. Ovary erect, 5-carpellate. Capsule linear, 1.5–3 cm long, seeds 3–6, ca. 3 mm long.

##### Distribution and habitat.

Yunnan; Sichuan; Guizhou; Guangxi. The species grows on damp ground near hills and streams in the understorey at an elevation of 800–1500 m.

##### Phenology.

Flowering and fruiting occur from July to November.

##### Additional specimens.

**China. Guizhou**: Guiyang City, *Yuanxin Xiong 86009* (PE); Zhenfeng County, *Tsiang 4512* (PE); Wangmo County, *Gongfan Wang 1-0142* (PE); Cengheng County, Fengcai Wang (PE); Renhuai City, *Lijiao Lou MT571* (ZY); Zhijin Country, *Fang Cheng 522425150910005LY* (GZTM). **Guangxi**: Nandan County, *Shengxiang Yu 3709* (PE). **Hunan**: *Daike Tian et al.*, *LS-2677* (CSH). **Yunnan**: Dali City, Qiwu Wang *63390* (PE); Luquan County, *Yongjie Guo 10CS1920* (KUN); Yangbi County, *Renchang Qin 25280* (KUN,PE); Weixin County, *Spice Plant Investigation Team 870552 & 870553* (KUN); Songming County, *Y.P.Chang 0130* (PE). **Sichuan**: Chengdu City, *C.Y.Wang 7469 & 7446* (PE); *Wenpei Fang 13140 & 13701* (KUN); Emeishan City, *Jihua Xiong et al.*, *33487 & 33574* (IBK,PE); *Shengxiang Yu 3274* (PE); *Hsiung et al.*, *33574* (IBSC); *Xiaojie Li LiXJ175* (KUN); *Zhongwu Yao 3361* (PE); Caiqi Li *3998* (PE); *Sichuan Plants*, *Western Academy of Sciences 891* (PE); *Gensheng Zhou 81205* (PE); *Zhengyi Wu 6439* (KUN); *T.N.Liou & C.Wang 797* (PE); *Cehong Li 96-39* (PE); *Chenglie Zhou 7267* (PE); Mabian Country, *Dejun Yu 4176 & 4175* (PE); *Deyuan Hong et al.*, *s.n.* (PE); Dujiangyan City, Wenpei Fang *6046* (PE).

## Supplementary Material

XML Treatment for Impatiens procumbens
